# Neonatal testosterone exposure alleviates female-specific severity of formalin-induced inflammatory pain in mice

**DOI:** 10.3389/fncir.2025.1593443

**Published:** 2025-07-02

**Authors:** Moeko Kanaya, Yoshifumi Ueta, Makiko Mochizuki-Kashio, Ayako Nakamura-Ishizu, Mariko Miyata

**Affiliations:** ^1^Division of Neurophysiology, Department of Physiology, School of Medicine, Tokyo Women's Medical University, Tokyo, Japan; ^2^Graduate School of Science and Engineering, Saitama University, Saitama, Japan; ^3^Department of Microscopic and Developmental Anatomy, Graduate School of Medicine, Tokyo Women's Medical University, Tokyo, Japan

**Keywords:** organizational effects, Gonadal hormones, formalin test, masculinization, BNST, PAG, microglia, T cell

## Abstract

Gonadal hormones may influence higher pain sensitivity in females than males by transiently activating the central pain pathway and organizing sexually dimorphic neuronal circuits during development. The latter effects of gonadal hormones, called organizational effects, are critical for establishing sex-specific reproductive functions and transforming them postnatally. However, it remains unclear whether the organizational effects determine sex-specific pain severity in adulthood. In this study, testosterone administration to female mice on day of birth alleviated intraplantar formalin injection-induced inflammatory pain in adulthood, resulting in comparable severity to males. In contrast, intense pain persisted in females with adult testosterone administration. We found no sex differences in thermal pain responses and spinal reflexes. Formalin injection similarly increased c-Fos activity in the spinal dorsal horn in both sexes, suggesting the involvement of supraspinal mechanisms and/or immune responses in sex-specific inflammatory pain. In the periaqueductal gray (PAG) region related to the descending pain modulation pathway, formalin increased c-Fos-positive cells in the lateral region of males but not females. In the bed nucleus of the stria terminalis (BNST) related to affective pain responses, formalin increased c-Fos-positive cells in females. Notably, in common with these regions, testosterone administration to neonatal females changed formalin-induced c-Fos activity from the female to the male type. We further examined the involvement of immune cells. Systemic microglial ablation using PLX3397 suppressed formalin-induced pain in a sex-independent manner. Although formalin injection changed T lymphocyte subsets in the peripheral blood in females, it was independent from neonatal testosterone administration. Therefore, the organizational effects of testosterone determine the male characteristic of formalin-induced inflammatory pain, possibly via sexually dimorphic PAG and BNST functions.

## 1 Introduction

Females generally experience a higher incidence of pain-related symptoms and exhibit greater pain sensitivity than males (Ghazisaeidi et al., 2023; Gregus et al., 2021; Mogil et al., 2024). In rodents, as in humans, females display increased sensitivity to both acute and chronic pain (Mogil, 2020). Gonadal hormones are a potential factor contributing to sexual dimorphism in pain perception (Craft, 2007). Testosterone and estrogen influence the brain and behavior via organizational and activational effects during the lifespan (Arnold and Breedlove, 1985; Nugent et al., 2012). Organizational effects occur during the development and permanently change the formation of the central nervous system, manifesting as functional disparities observed in adulthood. Activational effects transiently modulate neuronal activity and circuit function throughout life, depending on hormonal fluctuation or imbalance during life stages such as menstrual and menopause in females (Craft, 2007; Kundakovic and Rocks, 2022). Gonadal hormones affect inflammatory pain sensitivity through activational effects. For instance, estradiol administration increases pain responses in male rats following subcutaneous formalin injection (Aloisi and Ceccarelli, 2000). Castration of adult male rats prevents the usual decrease in pain responses to repeated formalin injections, while intact males show a reduction in pain sensitivity over time (Ceccarelli et al., 2003). These findings suggest that activational effects of gonadal hormones exert either pronociceptive or antinociceptive actions in formalin-induced inflammatory pain. However, it is still unclear what determines the sex-specific nature of pain sensitivity. The organizational effects of gonadal hormones are probably key to the formation of sex differences in the severity of inflammatory pain.

Sexual dimorphism in brain functions and behaviors is thought to arise from the sexually dimorphic organization of the central nervous system (Morris et al., 2004). Brain regions such as the anteroventral periventricular nucleus of the hypothalamus and the principal nucleus of the bed nucleus of the stria terminalis (BNST) are sexually dimorphic nuclei related to reproductive functions. Both nuclei differ in cell number and areal size between the sexes (Hines et al., 1992; Kanaya et al., 2014; Sumida et al., 1993). Regarding the lateral posterior BNST, males exhibit denser projections from the amygdala to this nucleus, a pattern observed in both mice and humans. This difference serves as an example of sex differences in the formation of fear memory (Florido et al., 2024). The BNST modulates pain sensitivity by releasing corticotropin-releasing factor (CRF) from neurons in the anterolateral subdivision (Ide et al., 2013). Female mice have larger CRF neurons in the anterolateral BNST than male mice (Uchida et al., 2019). Dopaminergic projection from the periaqueductal gray (PAG) to the BNST, which preferentially targets the dorsal part, including the anterolateral subdivision (Gungor and Pare, 2016), drives pain-related behaviors differently between male and female mice (Yu et al., 2021b). These observations indicate that PAG and BNST circuits are critical for generating the sexually dimorphic pain severity. Therefore, organizational effects of gonadal hormones during development may influence the severity of sex-specific pain by changing neuronal and circuit structures in these pain-related nuclei.

Neuroimmune mechanisms are another critical factor regulating sex differences in pain sensitivity. In the spinal cord, the roles of microglia and T lymphocytes are likely sexually dimorphic in neuropathic pain (Gregus et al., 2021; Mapplebeck et al., 2016; Midavaine et al., 2021). Peripheral nerve injury induces mechanical hypersensitivity in male mice through microglia-dependent mechanisms, whereas in female mice, T lymphocytes play a dominant role in its development (Gregus et al., 2021; Mapplebeck et al., 2016, 2018). Whether hormonal organizational effects produce this sex-specific dominance of microglia or T lymphocyte signaling in nociception is largely undetermined.

In this study, we examined the effects of testosterone on female-specific pain severity evoked by intraplantar formalin injection. Exogenous administration of testosterone after birth, but not in adulthood, suppressed formalin-induced pain severity in female mice, becoming similar to that in male mice. After formalin injection, male mice showed an increase in c-Fos-positive cells in the ventrolateral and lateral PAG regions, which are associated with the descending pain modulation pathway. In contrast, female mice showed formalin-induced c-Fos activation in the BNST but not in the lateral PAG. Testosterone administration to neonatal female mice changed the formalin-induced c-Fos activity in these regions to the male-specific responses. On the other hand, microglial contribution to formalin-induced pain was similar between the sexes, even with neonatal testosterone administration. In the peripheral blood, formalin increased the populations of T lymphocytes and their CD4^+^ subsets in female mice with neonatal testosterone administration while decreased CD8^+^ T lymphocyte subsets in female mice, regardless of neonatal testosterone administration. In contrast, these changes in T lymphocytes were absent in male mice. Therefore, our results suggest that testosterone during the neonatal period is required to organize the male pattern of inflammatory pain sensitivity depending on postnatal masculinization of PAG and BNST functions. Moreover, the organizational effects of testosterone are unlikely to induce sexually dimorphic roles of microglia or T lymphocytes in inflammatory pain sensitivity.

## 2 Materials and methods

### 2.1 Animals

Adult male and female C57BL/6N mice for breeding were purchased from Sankyo Labo Service Corporation (Tokyo, Japan). Offspring derived from mating in our facility were housed with dams in the same cages until weaning on postnatal day 21. All animals were bred and housed in a room with a controlled temperature (22 ± 1°C) and humidity (50 ± 15%) with a 12-h light/12-h dark cycle (lights on: 08:00–20:00). All animal experimental procedures adhered to guidelines for the Animal Care and Use Committee of the Tokyo Women's Medical University and the Care and Use of Experimental Animals of Saitama University.

### 2.2 Neonatal testosterone administration

On the day of birth, female pups were injected subcutaneously with testosterone propionate (100 μg, dissolved in 0.02 mL sesame oil, T0028, Tokyo Chemical Industry Co., LTD, Tokyo, Japan). This dose (50 mg/kg body weight, bw) is sufficient to masculinize brain regions responsible for reproduction (Morishita et al., 2017). As a control, female pups were injected with a sesame oil vehicle.

### 2.3 Adult testosterone administration

To mimic the testosterone state of mature males in females, adult female mice were treated with testosterone after ovariectomy (Chanda and Mogil, 2006). First, ovariectomy was performed on 7- to 10-week-old mice. Second, 2 weeks after the ovariectomy, mice were implanted subcutaneously in the back of the neck with either a 4.5-mm-long silicone tube (1.57 mm ID, 3.18 mm OD; Dow Corning, Midland, MI, USA) containing testosterone (T1500, Sigma-Aldrich, St. Louis, MO, USA) or an empty silicone tube. The size of this tube was determined based on the previous study, which demonstrated that it reproduces testosterone levels comparable to those found in sexually mature males (Wersinger et al., 1999). These surgical procedures were conducted under anesthesia using an isoflurane/oxygen gas mixture (2% for induction, 1% for maintenance).

### 2.4 Formalin test

Individual mice were habituated for 30 min in the test acrylic cylinder (10 cm diameter and 15 cm height) just before experiments. Mirrors were placed around the cylinder (two on both sides and the one under the cylinder) to observe mouse behaviors from various directions. Intraplantar injection of formalin (5% in saline, 20 μL) to the left hindpaw using a 30-gauge needle evoked licking behavior, which was observed and video-recorded for 65 min, including pre-injection time (for 5 min). The duration of formalin-induced licking was measured every 5 min for 1 hour. Total licking duration was divided into early (0–10 min, phase 1) and late (10–60 min, phase 2) phases because formalin injection evokes a two-phased pain: phase 1 responses are evoked by activation of cutaneous nerve endings and, after a brief silent phase, phase 2 responses recover due to inflammation reactions (Tjolsen et al., 1992). Experimental conditions were masked in the video analysis.

### 2.5 Hot-plate test

The hot-plate test was used to examine acute thermal pain responses associated with supraspinal pain pathways. Mice were habituated for 30 min in a test acrylic cylinder on a hot plate that had not yet been heated. After the habituation period, the hot plate was set to either 48°C or 52°C for testing (MK-350B; Muromachi Kikai Co., Ltd, Tokyo, Japan). Mice showed hindlimb shaking, licking, and jumping as pain responses (Minett et al., 2011; Deuis et al., 2017). The latency of these behaviors was separately measured to evaluate the pain intensity.

### 2.6 Tail-flick test

The tail-flick test was used to examine acute pain responses associated with spinal reflexes (Irwin et al., 1951) using the MK330B apparatus (Muromachi Kikai Co.). Mice were habituated to the experimenter through gentle handling for 1 week before the test began and to the test room for 30 min just before the test. The tail at distances of 2 cm, 3 cm, and 4 cm from the tip was exposed to a focused beam of light from a 50-W projection bulb (Bannon and Malmberg, 2007). The beam intensity scale was adjusted to 50 and 70, generating temperatures of 80 and 135°C, respectively. The tail-flick latency was measured to evaluate the pain intensity.

### 2.7 Microglial depletion by PLX3397 administration

To examine the contribution of microglia to the formalin-induced inflammatory pain, we used PLX3397 (also known as pexidartinib), a blood-brain barrier permeable inhibitor for the colony-stimulating factor 1 receptor and c-Kit, to deplete microglia from the brain and spinal cord (Elmore et al., 2014; Spiller et al., 2018). Mice were fed with a pellet diet containing PLX 3397 (PLX, 290 mg/kg, S7818, Selleckchem, Houston, TX, USA; or C1271, Chemgood, Glen Allen, VA, USA) to delete microglia for 5–7 days before the formalin test. Control mice were fed a standard pellet diet.

### 2.8 Immunohistochemistry

After 90 min from the saline or formalin intraplantar injection, mice were deeply anesthetized with a mixture of medetomidine hydrochloride (0.75 mg/kg bw, Domitor, Nippon Zenyaku Kogyo Co., Fukushima, Japan), midazolam (4 mg/kg bw, SANDOZ, Sandoz, Yamagata, Japan), and butorphanol tartrate (5 mg/kg bw, Vetorphale, Meiji Seika Pharma Co., Tokyo, Japan) and transcardially perfused with the fixative solution (4% paraformaldehyde in 0.1 M phosphate buffer (PB), pH 7.4). The spinal cords and brains were post-fixed in the fresh fixative at 4°C overnight, followed by equilibration to 30% sucrose in 0.1 M PB at 4°C for 2–3 days.

Fixed spinal cords were coronally cut into six parallel series of sections at a thickness of 50 μm using a cryostat (CM1860, Leica Biosystems, Wetzlar, Germany). One series of sections containing the lumbar 4–5 level (L4–5, 15–20 sections per series) were used for double immunofluorescence staining of calbindin-D28K to identify laminae I/II and c-Fos to assess neuronal activation following the formalin treatment. Sections were incubated in 5% normal donkey serum in 0.05 M phosphate-buffered saline (PBS) containing 0.3% Triton X-100 for 1 h at room temperature, followed by overnight incubation at 4°C with a mixture of a guinea pig polyclonal antibody against calbindin-D28K (1:1,000, MSFR100430, Nittobo Medical Co., LTD., Tokyo, Japan) and a rabbit monoclonal antibody against c-Fos (1:5,000, 226 003, Synaptic Systems, Göttingen, Germany) in 0.05 M PBS with 0.3% Triton X-100. The following day, after washing with PBS, the sections were incubated with a mixture of a donkey anti-guinea pig secondary antibody conjugated to Alexa Fluor 647 (1:500, 706-605-148, Jackson ImmunoResearch, West Grove, PA, USA) and a donkey anti-rabbit secondary antibody conjugated to Alexa Fluor Plus 488 (1:500, A21206, Thermo Fisher Scientific, Waltham, MA, USA) for 2 h at room temperature. After washing with PB, the sections were cover-slipped using CC/mount medium (Diagnostic BioSystems, Pleasanton, CA, USA).

For brain tissue, fixed brains were coronally sectioned at a thickness of 50 μm using a cryostat or a vibratome (VT1000S, Leica Biosystems). Serial sections containing the PAG and BNST were immunostained for neuronal nuclei (NeuN, a pan-neuronal marker), c-Fos, and vesicular glutamate transporter type 2 (VGluT2). Sections were reacted with a mixture of following primary antibodies: a mouse monoclonal antibody against NeuN (1:500, MAB377, Merck KGaA, Darmstadt, Germany), a rabbit monoclonal antibody against c-Fos (Synaptic Systems), and a guinea pig polyclonal antibody against VGluT2 (1:500, VGluT2-GP-Af810, Frontier Institute Co., Ltd, Ishikari, Japan). After washing with PBS, the sections were further incubated with secondary antibodies conjugated to Alexa Fluor 647 (1:500, 706-605-148, Jackson Immuno Research, for VGluT2), Alexa Fluor Plus 488 (Thermo Fisher Scientific, for c-Fos), and Alexa Fluor Plus 405 (1:500, A48257, Thermo Fisher Scientific, for NeuN). After washing with PB, the sections were cover-slipped using SlowFade Diamond antifade mountant (Thermo Fisher Scientific).

### 2.9 Images and data analysis

In the spinal cord L4–5 sections, c-Fos-positive cells were counted on the left side of the dorsal horn, ipsilateral to the saline or formalin intraplantar injection. Laminar I/II was identified using the intense calbindin-D28K immunoreactivity. Laminae I/II c-Fos-positive cells were manually counted across 7–9 sections in individual mice. In the spinal cord and brain regions, including the PAG and anterior BNST, the density of c-Fos-positive cells was quantified in the ipsilateral and contralateral sides of the formalin or saline injection using 3–4 sections in individual mice. Images were acquired using an epifluorescence microscope (BZ-X810, Keyence Corp., Osaka, Japan) and analyzed using ImageJ software (version 1.54g, https://imagej.nih.gov/ij). The contrast and brightness of acquired images were linearly adjusted for presentation using Adobe Photoshop CC software (Creative Cloud, Adobe Inc., San Jones, CA, USA).

### 2.10 Fluorescence-activated cell sorting (FACS) analysis of T lymphocytes

Peripheral blood samples were collected from the orbital plexus of mice using sterile hematocrit capillaries and transferred to EDTA-coated collection tubes at 90-min post-saline or formalin-injection. Collected blood samples were immediately lysed in the freshly prepared 1 × lysis buffer, consisting of 140 mM NH_4_Cl, 10 mM NaHCO_3_, and 1 mM EDTA, at room temperature and centrifuged at 300 × x g for 5 min at room temperature. The pellets were washed and resuspended in the FACS buffer (2 mM EDTA and 2% fetal bovine serum in PBS) containing a monoclonal antibody against CD16/32 (Clone 93, BioLegend Inc., San Diego, CA, USA) to block CD16/32 interactions with the Fc domain of immunoglobulins. The cells were then incubated with a panel of antibodies (phycoerythrin (PE)-anti-mouse CD3, Clone 17A2, BioLegend; PerCP/Cy5.5-anti-mouse CD4, Clone RM4-5, BioLegend; FITC-anti-mouse CD8a, Clone 53-6.7, BioLegend; APC-Cy7-anti-mouse CD45, Clone 30-F11, BioLegend) for 30 min at 4°C. After washing with PBS, cell surface immunolabeling was assessed using a CytoFLEX flow cytometer (Beckman Coulter Inc., Pasadena, CA, USA) and analyzed using FlowJo^TM^ software (FlowJo LLC, Ashland, OR, USA).

### 2.11 Statistical analysis

All statistics were performed using GraphPad Prism software version 10 for Mac (GraphPad Software, San Diego, CA, USA). Data are presented as the mean ± standard error of the mean (SEM). A repeated two-way analysis of variance (ANOVA) was used to evaluate the interaction between the main effects of a formalin test, such as elapsed time from saline or formalin administration, and sex-dependent pain responses. A one-way ANOVA was used to evaluate the sex-dependent pain responses in phases 1 and 2 of a formalin test, as well as in a hot-plate test and tail-flick test. An unpaired t-test was used to analyze differences in c-Fos expression in the spinal cord, PAG, and BNST between saline and formalin administration. An unpaired t-test was also used to evaluate the formalin-induced licking behavior in phases 1 and 2 with or without microglial depletion using PLX. An unpaired t-test was also used to analyze sex-dependent differences in proportions of peripheral T cell populations.

## 3 Results

### 3.1 Neonatal, but not adult, testosterone administration alleviates formalin-induced pain behavior in female mice

**Figure 1:**
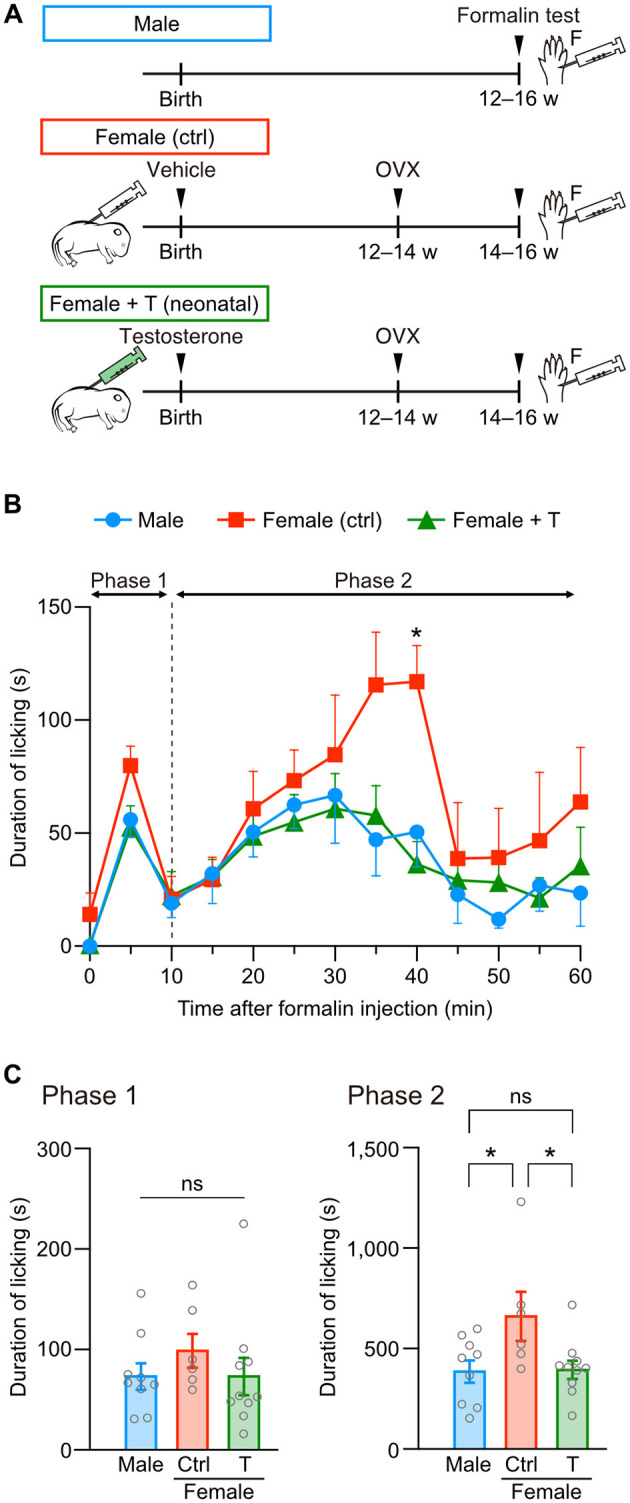
Neonatal testosterone administration alleviates formalin-induced pain behavior in female mice. **(A)** Time schedule for the experiment. Female pups were subcutaneously injected with testosterone (Female + T) or sesame oil vehicle (Female [ctrl]) on the day of birth. F, formalin intraplantar injection into the left hindpaw. **(B)** Time course of the licking duration after intraplantar formalin injection. Data are obtained from males (9 mice, blue circles), control females (6 mice, red squares), and testosterone-injected females (10 mice, green triangles). *p < 0.05 compared to both male and testosterone-injected female mice (Tukey's multiple comparisons test). Data are presented as the mean and SEM. **(C)** Comparisons of the duration of licking behavior during phases 1 and 2. Data are presented as the mean ± SEM. *p < 0.05 (Tukey's multiple comparisons test). ns, not significant.

We examined whether hormonal sexual manipulation is critical for organizing female-specific pain sensitivity using testosterone injection in neonatal and adult female mice. First, on the day of birth, female pups underwent subcutaneous injection with testosterone or a sesame oil vehicle as control. Later, at postnatal 12–14 weeks, these female mice underwent ovariectomy. After 2 weeks from the ovariectomy, we performed a formalin test using these female and age-matched male mice (Figure 1A). Intraplantar formalin injection into the left hindpaw biphasically evoked pain responses like licking of the hindpaw. Direct injury of cutaneous sensory fibers and inflammatory reactions evoke licking responses in the early (phase 1, 0–10 min) and late phases (phase 2, 10–60 min), respectively (Figure 1B). Formalin injection increased the total duration of licking behavior in phases 1 and 2 compared to saline injection in male, female control, and female mice with neonatal testosterone administration (female + T), indicating formalin-induced pain among groups (Supplementary Figures 1A–C). On the other hand, the intensity of formalin-induced pain was different depending on the sex or neonatal testosterone exposure. The total licking duration in phase 1 was similar among groups (Figure 1C). Consistent with previous studies (Aloisi et al., 1994; Perissin et al., 2003), phase 2 responses were more significant in female control than male mice. However, female mice with neonatal testosterone administration showed suppressed pain behavior at phase 2, similar to male mice (Figure 1C). These results indicate that neonatal testosterone exposure can masculinize inflammatory pain responses in female mice.

**Figure 2:**
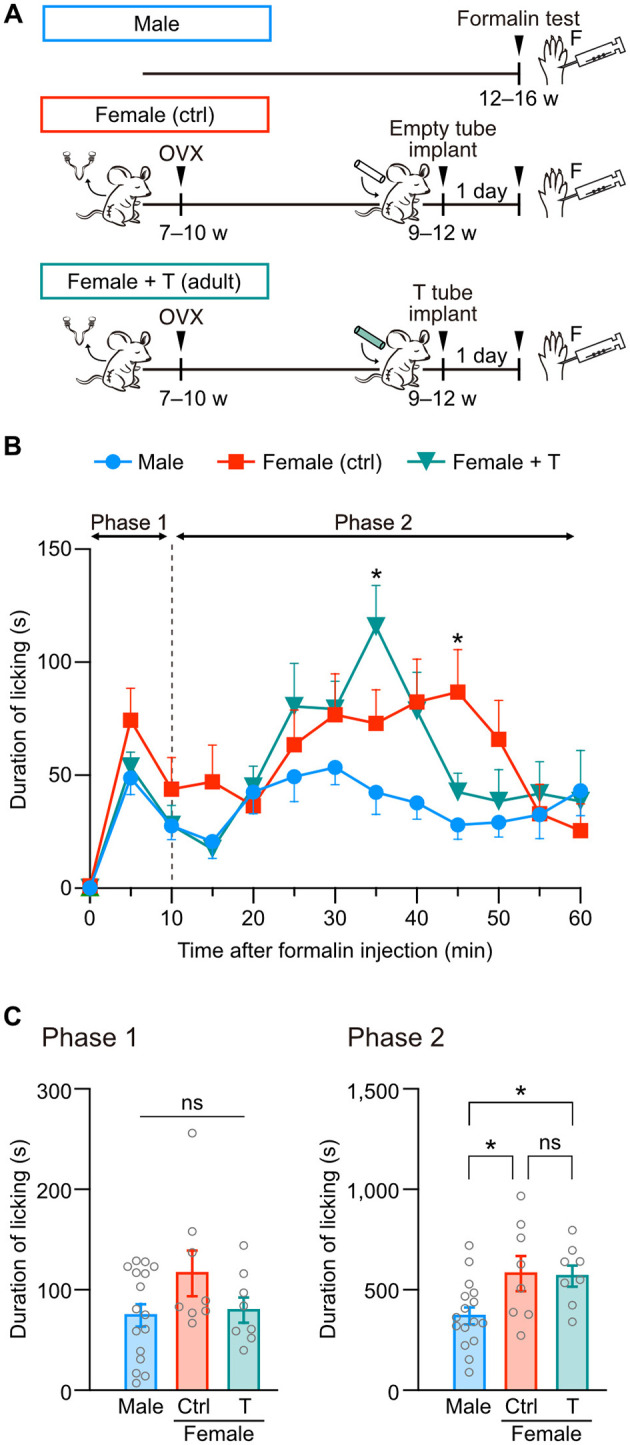
Adult testosterone administration maintains formalin-induced pain behavior in female mice. **(A)** Time schedule for the experiment. Adult female mice were subcutaneously implanted with a tube containing testosterone (Female + T) or an empty tube (Female [ctrl]) 2 weeks after the ovariectomy (OVX). **(B)** Time course of the licking duration after intraplantar formalin injection. Data are obtained from males (16 mice, blue circles), control females (8 mice, red squares), and testosterone tube-implanted females (8 mice, green triangles). *p < 0.05 compared to male mice (Tukey's multiple comparisons test). Data are presented as the mean and SEM. **(C)** Comparisons of the duration of licking behavior during phases 1 and 2. Data are presented as the mean ± SEM. *p < 0.05 (Tukey's multiple comparisons test). ns, not significant.

Next, we examined whether testosterone exposure in adulthood can change the severity of formalin-induced pain in female mice. A silicon tube containing testosterone or an empty tube was implanted at the back of the neck in female mice after ovariectomy (Figure 2A). The total licking duration in phase 1 was similar among groups (Figures 2B, C). In contrast to neonatal testosterone exposure, the total licking duration in phase 2 was significantly longer in female mice with testosterone tube implantation than in male mice (Figure 2C). These results indicate that adult testosterone exposure in female mice failed to suppress pain behavior (Figure 2C). Therefore, the gonadal hormone milieu during the neonatal period rather than the testosterone levels at the time of a formalin test is critical for organizing sex-specific inflammatory pain responses in adulthood.

### 3.2 Sex difference is absent in thermal pain and spinal dorsal horn activation

**Figure 3:**
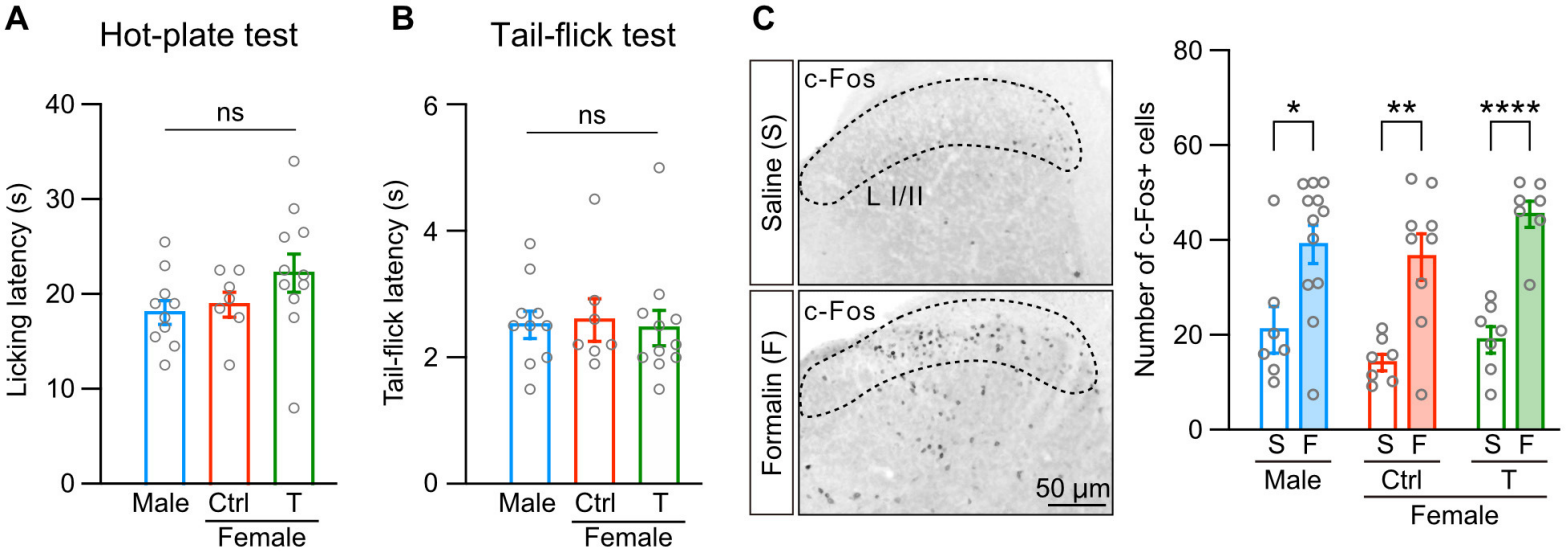
Sex difference is absent in thermal pain and spinal dorsal horn activation. **(A)** Licking latency in a hot-plate test (52°C) in males (10 mice, blue bar), control females (Female [ctrl], 7 mice, red bar), and females with neonatal testosterone administration (Female + T, 11 mice, green bar). Data are presented as the mean ± SEM. ns, not significant. **(B)** Tail-flick latency in a tail-flick test (beam intensity generating heart temperature at 135°C). Data are obtained from the same populations in **(A)**. Data are presented as the mean ± SEM. ns, not significant. **(C)** c-Fos immunoreactivity (left images) and the number of c-Fos positive cells in laminae I/II (L I/II) of the lumber cord in response to saline **(S)** or formalin **(F)** injection into the left hindpaw (right graphs). Data are obtained from the same populations in **(A)**. *, p <0.05; **, p <0.01; and ****, p <0.0001 (unpaired t-test).

In contrast to inflammatory pain, sexual dimorphism in thermal pain is still controversial in rodents (Chesler et al., 2002; Mogil et al., 2000). Therefore, we examined the effect of neonatal testosterone exposure on thermal pain. We found that neither licking latencies in a hot-plate test nor tail-flick latencies in a tail-flick test were different among male, female control, and female mice with neonatal testosterone administration (Figures 3A, B). In addition, we found no sex differences in shaking or jumping responses in a hot-plate test (Supplementary Figure 2A). Pain responses depending on the intensity of thermal stimulation also showed no sex differences in hot-plate and tail-flick tests (Supplementary Figures 2A, B). These results indicate the absence of apparent sex differences in thermal pain.

Equivalent tail-flick reflexes between males and females may suggest that thermal stimulation activates spinal circuits in a sex-independent manner. Intraplantar formalin injection also activates spinal dorsal horn neurons and nociceptive processing in the spinal cord (Hirota et al., 2023; Zhao et al., 2007). Therefore, we examined formalin-induced spinal dorsal horn activities using c-Fos immunohistochemistry. Formalin injection increased c-Fos-positive cells in calbindin-D28K-positive laminae I/II of the lumber cord compared to saline injection (Figure 3C and Supplementary Figure 2C). Formalin similarly increased c-Fos-positive cells among male, female control, and female mice with neonatal testosterone administration (Figure 3C). These findings suggest that sexual dimorphism in formalin-induced inflammatory pain may be caused by activity in supraspinal brain regions, inflammatory responses, or both.

### 3.3 Neonatal testosterone administration masculinizes female-specific formalin-induced c-fos activity in the PAG and BNST

**Figure 4:**
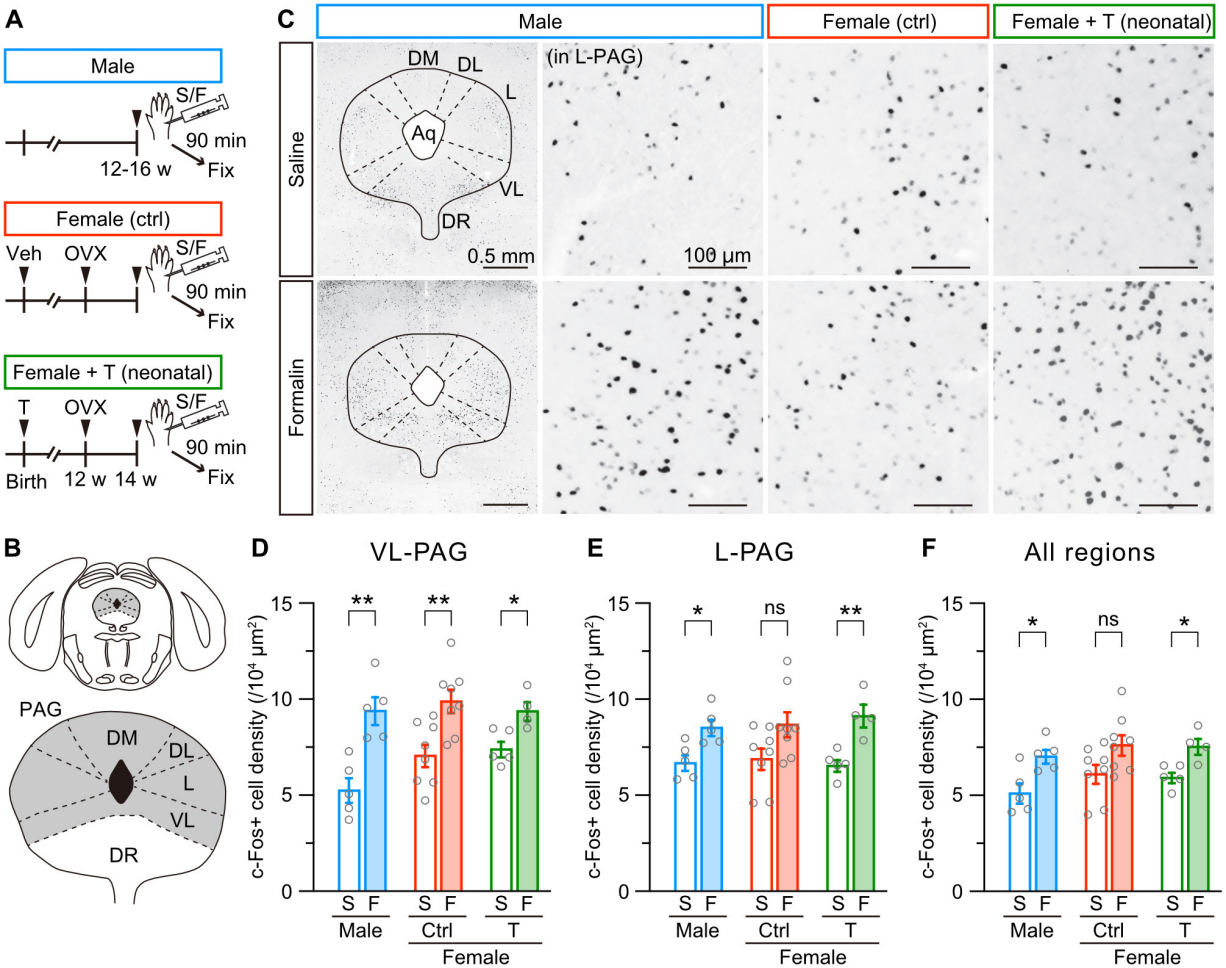
Neonatal testosterone administration masculinizes female-specific formalin-induced c-Fos activity in the PAG. **(A)** Time schedule for the experiment. Female pups were subcutaneously injected with testosterone (Female + T) or sesame oil vehicle (Female [ctrl]) on the day of birth. In adulthood, mice were sampled 90 min after saline **(S)** or formalin **(F)** injection. **(B)** Schematic representation of the analyzed PAG area divided into the dorsomedial (DM-PAG), dorsolateral (DL-PAG), lateral (L-PAG), and ventrolateral (VL-PAG) divisions. DR, dorsal raphe. **(C)** c-Fos immunoreactivity in the PAG. Magnified images were captured from the L-PAG. Aq, cerebral aqueduct. **(D–F)** The density of c-Fos positive cells in the VL-PAG, L-PAG, and all regions following saline or formalin injection into the left hindpaw. Data are obtained from males (2515 ± 217 cells in 5 mice for S and 3212 ± 142 cells in 5 mice for F), control females (2608 ± 187 cells in 8 mice for S and 3475 ± 221 cells in 8 mice for F), and females with neonatal testosterone administration (2570 ± 92 cells in 5 mice for S and 3776 ± 230 cells in 4 mice for F). *p < 0.05; and **p < 0.01 (unpaired t-test). ns, not significant. Data are presented as the mean ± SEM. Each circle corresponds to different mice.

Gonadal hormones are necessary to form sexually dimorphic brain nuclei, such as the BNST and hypothalamus (Kanaya et al., 2014; Shah et al., 2004). The anterior BNST is involved in affective behavior, including nociception, via afferent and efferent connections (Gungor and Pare, 2016). BNST neurons receive dopaminergic and glutamatergic inputs from the PAG (Zhao et al., 2022). At least, PAG dopaminergic inputs to BNST function differently between male and female mice during pain (Yu et al., 2021b). Based on these observations, we examined whether c-Fos activity in the PAG evoked by intraplantar formalin injection differs among male, female control, and female mice with neonatal testosterone administration (Figure 4A and Supplementary Figure 3A). The PAG comprises dorsomedial, dorsolateral, lateral, and ventrolateral divisions (Figure 4B and Supplementary Figure 3B). Formalin injection increased c-Fos-positive cells in the ventrolateral division similarly among all groups compared to saline injection In contrast, the formalin-induced increase of c-Fos-positive cells in the lateral division was significant in male and female mice with neonatal testosterone administration but not in female control mice (Figures 4C–E). We found a similar tendency of sex-specific c-Fos activity in the whole PAG (Figure 4F). Dorsomedial and dorsolateral divisions exhibited no apparent increase of c-Fos activity by formalin injection among all groups (Supplementary Figure 3C). These results indicate the sexual dimorphism of formalin-induced c-Fos activity in the PAG. Also, neonatal testosterone exposure can masculinize c-Fos activity in the PAG in inflammatory pain.

**Figure 5:**
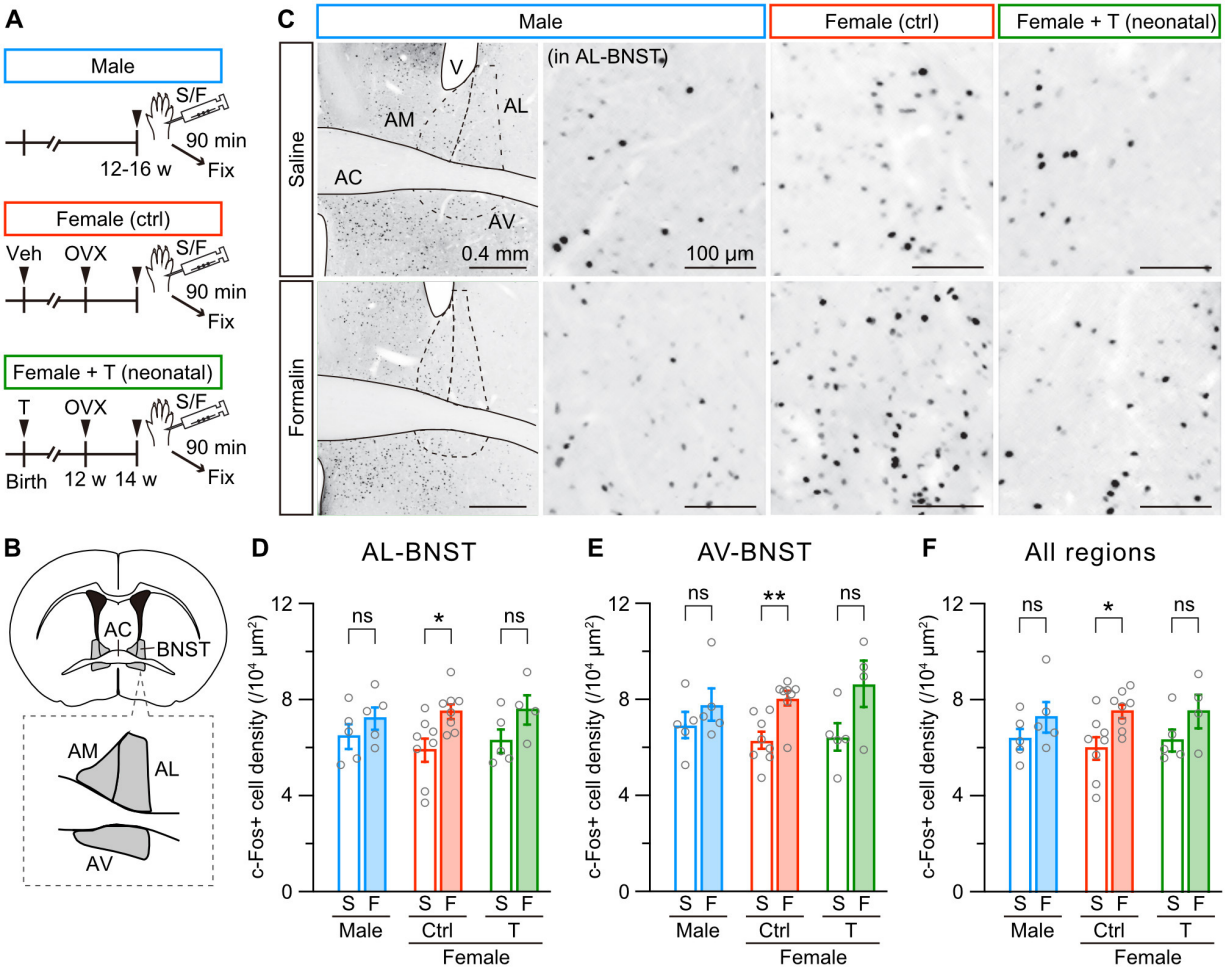
Neonatal testosterone administration masculinizes female-specific formalin-induced c-Fos activity in the BNST. **(A)** Time schedule for the experiment. Female pups were subcutaneously injected with testosterone (Female + T) or sesame oil vehicle (Female [ctrl]) on the day of birth. In adulthood, mice were sampled 90 min after saline (S) or formalin **(F)** injection. **(B)** Schematic representation of the analyzed anterior BNST area divided into the anteromedial (AM-BNST), anterolateral (AL-BNST), and anteroventral (AV-BNST) divisions. AC, anterior commissure. **(C)** c-Fos immunoreactivity in the anterior BNST. Magnified images were captured from the AL-BNST. V, lateral ventricle. **(D–F)** The density of c-Fos positive cells in the AL-BNST, AV-BNST, and all regions following saline or formalin injection into the left hindpaw. Data are obtained from males (1388 ± 147 cells in 5 mice for S and 1719 ± 240 cells in 5 mice for F), control females (1139 ± 66 cells in 8 mice for S and 1665 ± 164 cells in 8 mice for F), and females with neonatal testosterone administration (1339 ± 78 cells in 5 mice for S and 1900 ± 210 cells in 4 mice for F). *p < 0.05; and **p < 0.05 (unpaired t-test). ns, not significant. Data are presented as the mean ± SEM. Each circle corresponds to different mice.

We further examined formalin-induced c-Fos activity in the anterior BNST among male, female control, and female mice with neonatal testosterone administration (Figure 5A and Supplementary Figure 3A), which comprises dorsal (anteromedial and anterolateral) and ventral (anteroventral) divisions (Figure 5B and Supplementary Figure 3D). Formalin injection significantly increased c-Fos-positive cells in the anterolateral and anteroventral divisions but not in the anteromedial division of the BNST in female control mice. In male mice, formalin injection did not increase c-Fos expression in these regions (Figures 5C–E and Supplementary Figure 3E). The same tendency existed in the whole area of the BNST (Figure 5F). Neonatal testosterone administration was able to masculinize c-Fos activity in the BNST (Figures 5C–F). Therefore, the PAG and anterior BNST likely play a pivotal role in sex-specific inflammatory pain processing. Furthermore, neonatal testosterone exposure is thought to alleviate female-specific severity of inflammatory pain via masculinization of PAG and BNST structures and functions.

### 3.4 Microglial ablation similarly suppresses formalin-induced inflammatory pain responses between males and females

**Figure 6:**
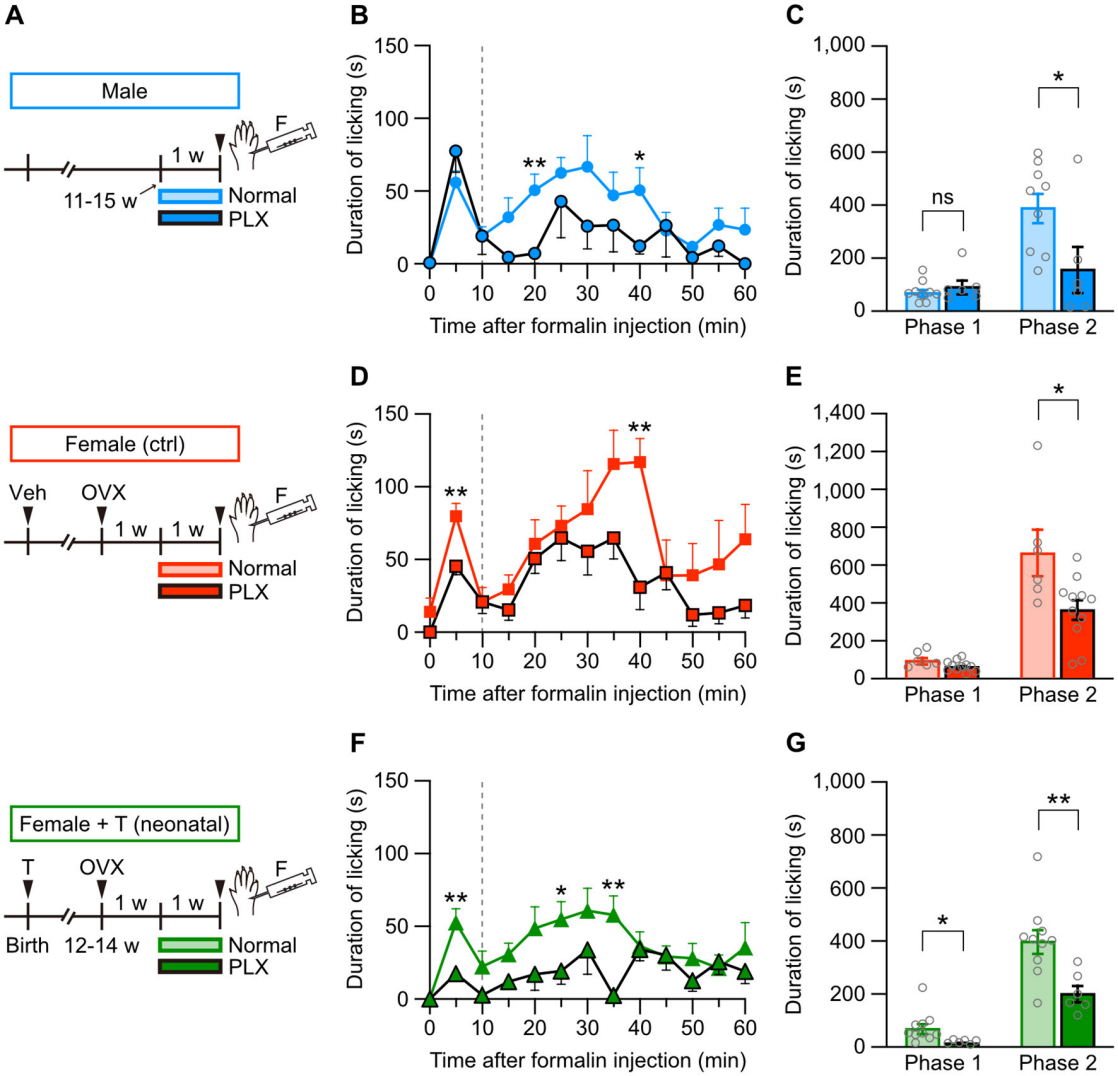
Microglial ablation suppresses formalin-induced pain responses in males and females. **(A)** Time schedule for the experiment. Female pups were subcutaneously injected with testosterone (Female + T) or sesame oil vehicle (Female [ctrl]) on the day of birth. In adulthood, males, control females, and females with neonatal testosterone administration were fed a PLX-containing diet (PLX, 290 mg/kg) or a normal diet for 1 week (5–7 days), followed by a formalin test. **(B)** Time course of the licking duration after intraplantar formalin injection in males (9 mice for normal and 6 mice for PLX). *p < 0.05; and **p < 0.01 compared to normal male mice (Tukey's multiple comparisons test). Data are presented as the mean and SEM. Data for normal males are the same as in Figure 1B. **(C)** Comparisons of the duration of licking behavior during phases 1 and 2. Data are presented as the mean ± SEM. *p < 0.05 (unpaired t-test). ns, not significant. **(D)** Time course of the licking duration after intraplantar formalin injection in control females (6 for normal and 11 for PLX). **p < 0.01 compared to normal female mice. Data for normal females are the same as in Figure 1B. **(E)** Comparisons of the duration of licking behavior during phases 1 and 2. *p < 0.05. **(F)** Time course of the licking duration after intraplantar formalin injection in females with neonatal testosterone administration (10 for normal and 6 for PLX). *p < 0.05; and **p < 0.01 compared to normal females with neonatal testosterone administration. Data for normal females with neonatal testosterone administration are the same as in Figure 1B. **(G)** Comparisons of the duration of licking behavior during phases 1 and 2. * p <0.05; and **p < 0.01.

Female-specific severity of formalin-induced, but not thermal, pain suggests a crucial role of inflammatory responses in sex-specific pain sensitivity (see Figures 1–3). Previous studies have indicated that the immune system, such as microglia or T lymphocytes, functions differently between male and female animals during nociception (Ghazisaeidi et al., 2023). Therefore, we first examined the contribution of microglia to formalin-induced inflammatory pain by using microglial ablation technique. PLX3397 (PLX) is an inhibitor for colony-stimulating factor 1 receptors, which mediates signaling required for microglial survival and maintenance. Administration of PLX-containing diet effectively depletes microglia in the brain and spinal cord (Elmore et al., 2014; Spiller et al., 2018; Ueta and Miyata, 2021). Before a formalin test, we administered a PLX-containing diet for 1 week (5–7 days) to male or female mice (Figure 6A). PLX administration started 1 week after the ovariectomy in female mice (Figure 6A). In male mice, PLX administration unchanged the licking duration in phase 1 but decreased the licking duration in phase 2 after intraplantar formalin injection (Figures 6B, C). Similarly, in female control mice, PLX moderately affected the licking duration in phase 1 but decreased the licking duration in phase 2 (Figures 6D, E). PLX decreased the licking duration in phases 1 and 2 in female mice with neonatal testosterone administration (Figures 6F, G). We found in phase 2 that the degree of PLX-dependent decrease of the licking duration was similar among groups (Supplementary Figure 4), suggesting sex-independent suppression of formalin-induced inflammatory pain responses under microglial ablation.

### 3.5 Formalin-induced changes in T lymphocyte subsets in female mice are devoid of masculinization by neonatal testosterone administration

**Figure 7:**
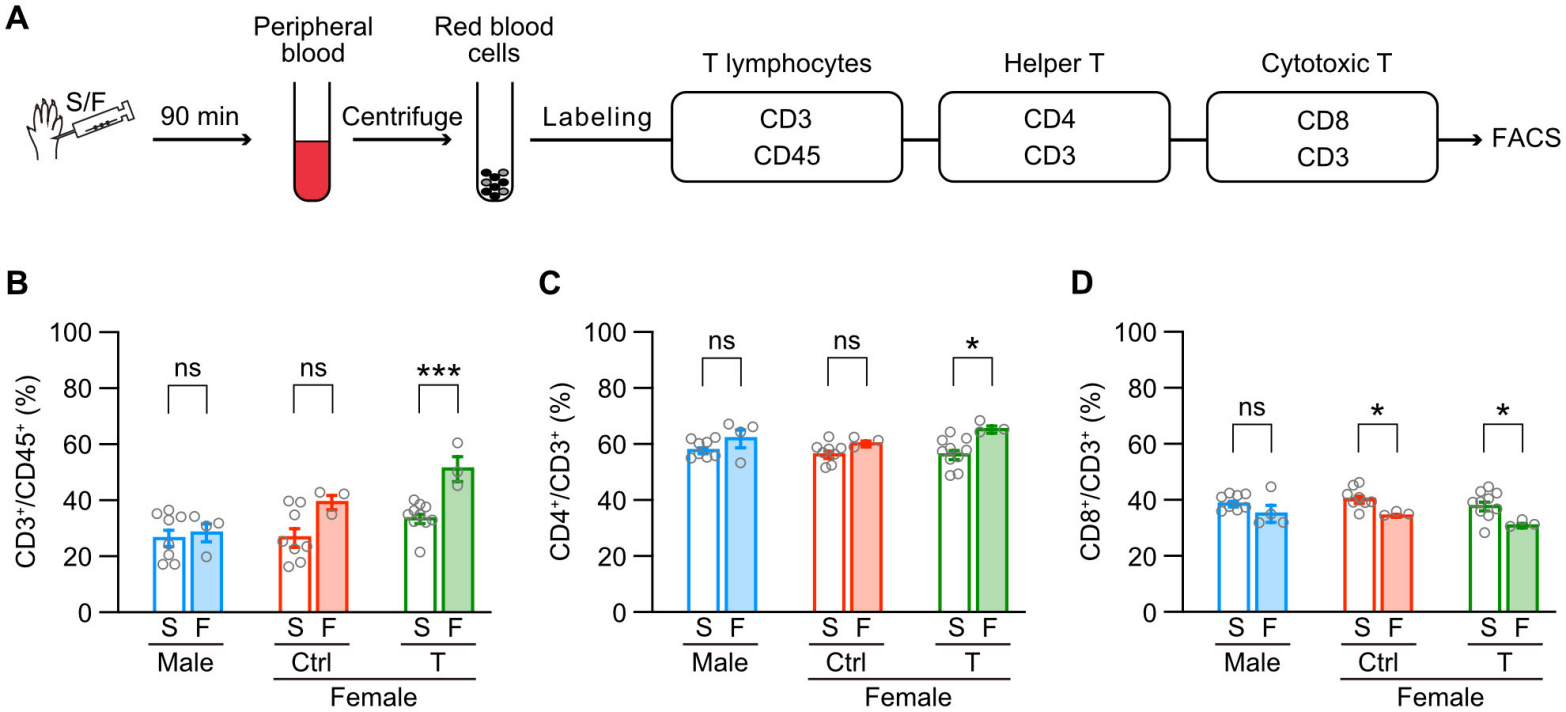
Testosterone's masculinizing effects are absent in formalin-induced behavior of peripheral T lymphocytes in females. **(A)** Schematic representation of the sample flow through the fluorescence-activated cell sorting (FACS) process. **(B)** Percentages of T lymphocytes (CD3+) among leucocytes (CD45+) in the peripheral blood. **(C)** Percentages of helper T lymphocytes (CD4+) among all T lymphocytes. **(D)** Percentages of cytotoxic T lymphocytes (CD8+) among all T lymphocytes. Data are obtained from males (8 mice for S and 4 mice for F), control females (Female [ctrl], 8 mice for S and 3 mice for F), and females with neonatal testosterone injection (Female + T, 10 mice for S and 3 mice for F). S, saline; F, formalin intraplantar injection into the left hindpaw. Data are presented as the mean ± SEM. *p < 0.05; and ***p < 0.001 (unpaired t-test). ns, not significant.

Finally, we examined whether intraplantar formalin injection changes subsets of T lymphocytes in the peripheral blood depending on the sex or neonatal testosterone administration (Figure 7A). Formalin injection significantly increased the proportion of CD3^+^ T lymphocytes in CD45^+^ leukocytes in female mice with neonatal testosterone administration but not in male and female control mice (Figure 7B). Formalin injection also increased the subset of CD4^+^/CD3^+^ helper T lymphocytes in female mice with neonatal testosterone administration (Figure 7C). In contrast, formalin injection decreased the subset of CD8^+^/CD3^+^ cytotoxic T lymphocytes in female mice with or without neonatal testosterone administration (Figure 7D). These results indicate that formalin-induced inflammation could change the behavior of peripheral T lymphocytes in female mice independent of the organizational effects of testosterone. Therefore, inflammatory responses via microglia in the central nervous system or T lymphocytes in the peripheral blood may be irrelevant to the masculinization of inflammatory pain in female mice caused by neonatal testosterone exposure.

## 4 Discussion

### 4.1 The organizational effects of testosterone masculinize inflammatory pain severity in female mice

The organizational effects of gonadal hormones are a prominent cause of sexually dimorphic behavior and functions in animals. Over half a century ago, pioneering studies described the critical role of testosterone during the developmental sensitive period in male and female sexual behavior in rats. Castration within 1 week after birth induces feminization and demasculinization of sexual behavior, increasing lordosis posture in response to male mounting (Feder and Whalen, 1965). Similarly, male rats castrated within 1 week after birth and administered testosterone in adulthood show sexually motivated but incomplete mating sequences (Hart, 1968). Conversely, testosterone administration to perinatal female rats induces defeminization and masculinization of sexual behavior, exhibiting a male pattern of copulatory behavior (Gladue and Clemens, 1982; Sachs et al., 1973). Neonatal testosterone administration also induces sterility and ovarian malfunction in female rats (Barraclough, 1961; Smith and Peng, 1966). These observations indicate that the organizational effects of testosterone permanently change adult reproductive functions.

Hormonal organizational effects are likely essential for sex-specific pain-related functions in rodents. Female rats display significantly lower morphine analgesic effects than male rats. However, testosterone administration in female rats during development increases morphine analgesic effects comparable to male rats (Krzanowska et al., 2002). Non-opioid analgesics induced by environmental stress (for example, swimming in cold water) are also sexually dimorphic in mice, depending on the testosterone level during the early developmental period (Mogil et al., 1993; Sternberg et al., 1995). Females exhibit higher anxiety-like behavior induced by physiological stress, but this sex difference can be eliminated by administering gonadal hormones to females during development (Wright et al., 2023). In addition, our results indicate that testosterone administration during the neonatal period, but not in adulthood, masculinizes female-specific severity of formalin-induced inflammatory pain in female mice (Figures 1, 2). Therefore, like reproductive functions, organizing pain-related functions is highly sensitive to gonadal hormones during development. These organizational effects may change the fate of neurons and synapses in the hormonal-sensitive period, like the critical or sensitive period in the sensory and limbic systems (Reh et al., 2020), to induce differentiation of sexually dimorphic neuronal structures (Bangasser and Cuarenta, 2021; Mikulovic and Lenschow, 2025; Morris et al., 2004).

### 4.2 Supraspinal mechanisms underlying sexual dimorphism in inflammatory pain sensitivity

The ventrolateral PAG is an essential component of the descending inhibitory pain pathway (Bagley and Ingram, 2020; Lau and Vaughan, 2014). Stimulation of the ventrolateral PAG reduces the pain response to formalin injection (Dennis et al., 1980). Descending axons from the ventrolateral PAG terminate and corelease dopamine and glutamate in the dorsal sector of the anterior BNST. The dopaminergic projections from the ventrolateral PAG to the BNST induce antinociceptive action in male mice but not in female mice, whereas these projection promote locomotion in females (Li et al., 2016; Yu et al., 2021b). In addition, glutamatergic neurons in the lateral PAG provide excitatory inputs to noradrenaline neurons in the locus coeruleus (Barcomb et al., 2022), which originate descending pathways involved in pain suppression (Llorca-Torralba et al., 2016). Consistent with these observations, a significant increase of c-Fos-positive neurons in the lateral PAG in male and masculinized female mice (Figure 4E), along with lower pain behavior in response to formalin injection, likely reflects sex differences in the descending antinociceptive modulation from the PAG to the BNST circuits. More importantly, our results suggest that neonatal testosterone level contributes to the development of sex differences in analgesic effects via descending analgesic pathways, including PAG-BNST. In the anterior BNST, formalin injection significantly increased c-Fos activity in female mice in the anterolateral and anteroventral subdivisions (Figures 5D, E). CRF neurons in the BNST (Daniel and Rainnie, 2016), which are more abundant in females (Uchida et al., 2019), influence multiple facets of the pain experience and impact the sex-specific expression of pain-related behaviors (Yu et al., 2021a). In addition, CRF signaling in the anterolateral BNST contributes to the emotional components of pain (Ide et al., 2013). The anteroventral BNST also mediates painful emotion (Deyama et al., 2008). Based on these observations, formalin-induced activation of CRF neurons possibly increases anxiety levels, leading to an escalation of pain-related behaviors in female mice. Therefore, a formalin-induced activity of anterolateral and anteroventral BNST neurons might enhance the affective component of pain in female mice. Neonatal testosterone level may also contribute to developing these female-specific functions that control the emotional and stressful aspects of pain in the anterior BNST.

Our findings propose that the sexually dimorphic organization of the PAG and BNST is susceptible to neonatal testosterone exposure during development. Both areas abundantly express androgen and estrogen receptors (Simerly et al., 1990). Therefore, sex differences in inflammatory pain responses may be caused by the activity of supraspinal mechanisms such as descending pain modulation pathways via the PAG and the limbic system controlling affective components of pain.

### 4.3 Involvement of immune systems in sex differences in inflammatory pain sensitivity

Roles of immune cells such as microglia and T lymphocytes in chronic neuropathic pain are sexually dimorphic in rodent models for peripheral nerve injury (Gregus et al., 2021; Mapplebeck et al., 2016; Midavaine et al., 2021). Mechanical hypersensitivity induced by spared nerve injury requires microglial activation in the spinal cord in male mice, while in female mice, T lymphocytes likely contribute to mechanical hypersensitivity (Sorge et al., 2015). Pannexin-1 channels are one potential mechanism underlying this sexual dimorphism in microglia and T lymphocytes because pannexin-1 drives microglia-dependent signaling in males and CD8^+^ T lymphocyte-dependent signaling in females to induce mechanical hypersensitivity (Fan et al., 2025). Colony-stimulating factor 1 released from the injured nerve activates microglia in the spinal cord of male mice. In contrast, it promotes the expansion of regulatory T lymphocytes within the spinal meninges of female mice (Kuhn et al., 2021). However, peripheral nerve injury induces microgliosis in male and female rats, although inhibiting microglia or purinergic signaling in the spinal cord attenuates mechanical hypersensitivity only in male rats (Mapplebeck et al., 2018). A study using mice indicates that mechanical hypersensitivity induced by spared nerve injury is associated with increased cell surface expression of P2X4 receptors in microglia both in males and females (Gilabert et al., 2023). Therefore, although the immune cell contribution to neuropathic pain differs between sexes, females could utilize microglia-dependent mechanisms to induce neuropathic pain like males.

Microglia critically contribute to formalin-induced inflammatory pain. Within one hour of the formalin stimulation, microglia in the spinal cord show increased process dynamics and outward-rectifying potassium currents evoked by depolarization. However, formalin-induced reactivity of these microglial morphological and electrophysiological properties shows no sex differences (Gu et al., 2022). Our results also indicate the contribution of microglia to formalin-induced inflammatory pain because microglial depletion using PLX3397 suppressed pain responses (Figure 6). The extent of pain suppression in phase 2 by microglial depletion was similar between the sexes (Supplementary Figure 4), suggesting the sex-independent contribution of microglia to acute inflammatory pain, unlike chronic neuropathic pain. Formalin injection increases the immunoreactivity of P2X4 receptors and the number of P2X4 receptor-positive microglia in the spinal dorsal horn of male rats moderately 1 day after the injection, reaching a peak until 7 days after the injection (Guo et al., 2005). Similarly, formalin injection induces long-lasting paw edema and long-term thermal and mechanical hyperalgesia in male rats (Fu et al., 2001). Aggravation of microgliosis in the spinal dorsal horn may be necessary for long-term hyperalgesia after formalin injection (Fu et al., 2000). These observations suggest that male-specific microglia-dependent signaling significantly contributes to long-term hyperalgesia rather than acute inflammatory pain responses after formalin injection.

In contrast to inflammatory pain responses during phase 2, PLX3397 application suppressed pain responses during phase 1 only in control and testosterone-injected female mice but not in male mice (Figure 6). Phase 1 pain response in a formalin test reflects peripheral nociceptor activation, suggesting that PLX3397 inhibits female-specific immune mechanisms regulating nociceptor activity. Recent transcriptome analyses report that mRNA translation in Nav1.8-positive dorsal root ganglion (DRG) neurons, most of which are nociceptor and also express transient receptor potential vanilloid type 1 (TRPV1) known as capsaicin receptor, is differently regulated between male and female mice (Tavares-Ferreira et al., 2022). TRPV1 activation is sexually dimorphic in neuropathic and formalin-induced inflammatory pain model mice. Peripheral nerve injury and formalin injection both drive macrophages in the DRG to release IL-23, which further induces IL-17A release from macrophages. TRPV1 in DRG neurons mediate these chemokines to produce mechanical hypersensitivity in female but not male mice (Luo et al., 2021). Systemic PLX3397 application effectively depletes macrophages in the DRG (Lund et al., 2024). Based on these observations, it is conceivable that PLX3397 application inhibits female-specific nociceptor activation dependent on macrophage-TRPV1 signaling, resulting in female-specific suppression of phase 1 pain responses. In addition, our results suggest that neonatal testosterone level is unrelated to organizing sexual dimorphism in macrophage-TRPV1 signaling in the DRG to produce mechanical hypersensitivity.

Previous studies suggest that T lymphocytes are unlikely to contribute to inflammatory pain in rodents. For example, genetic ablation of T cell receptor β-positive T lymphocytes, including such as CD4^+^ or CD8^+^ subsets, permits thermal and mechanical hypersensitivity induced by intraplantar injection of complete Freund's adjuvant or plantar incisional wound, similar to intact male mice (Ghasemlou et al., 2015). In addition, genetic ablation of T cell receptor δ-positive T lymphocytes, corresponding to γδT subsets usually negative for CD4 and CD8, also permits thermal and mechanical hypersensitivity evoked by intraplantar formalin injection in male and female mice (Petrovic et al., 2019). Intraperitoneal formalin injection recruits leukocytes and lymphocytes in the intraperitoneal cavity in female rats, probably releasing lipid mediators, cytokines, and/or chemokines. In the formalin test, peaks of formalin-induced increase of leukocytes and lymphocytes occur after 2–4 hours and 24 hours from the injection (Santos et al., 2004). Consistent with these reports, the formalin injection moderately elevated the number of T lymphocytes only in female mice with or without neonatal testosterone administration (Figure 7), suggesting that the contribution of T cell response to inflammatory pain may be an innate response in the female. Thus, we found that neonatal testosterone exposure contributes to the formation of sex differences in formalin-induced inflammatory pain behavior, in which the development of descending analgesic function via the PAG -BNST may be involved.

## Data Availability

The original contributions presented in the study are included in the article/Supplementary material, further inquiries can be directed to the corresponding author.

## Supplementary material

Supplementary Figure 1 Intraplantar formalin injection increases pain responses. **(A)** Male pain responses to intraplantar saline or formalin injection. Left graphs, the time course of the licking duration after intraplantar saline (5 mice) or formalin injection (9 mice). Right graphs, comparisons of the duration of licking behavior during phases 1 and 2. Data are presented as the mean ± SEM. Data for formalin-administered males are the same as in Figure 1B. ^*^*p* < 0.05; and ^**^*p* < 0.01 (left graphs, Tukey's multiple comparisons test; and right graphs, unpaired *t*-test). ns, not significant. **(B)** Female pain responses to intraplantar saline (5 mice) or formalin injection (5 mice). Data for formalin-treated females are the same as in Figure 1B. ^*^*p* < 0.05; ^**^*p* < 0.01; and ^***^*p* < 0.001. **(C)** Pain responses of females with neonatal testosterone administration to saline (5 mice) or formalin injection (10 mice). Data for formalin-treated mice are the same as in Figure 1B. ^*^*p* < 0.05; ^**^*p* < 0.01; and ^****^*p* < 0.0001.

Supplementary Figure 2 Thermal pain responses are similar among males, control females, and females with neonatal testosterone administration. **(A)** Latencies to different pain responses at 48°C (left graphs) and 52°C (right graphs) in a hot-plate test. Data are obtained from males (10 mice, blue bars), control females with neonatal sesame oil administration (Female [ctrl], 7 mice, red bars), and females with neonatal testosterone administration (Female + T, 11 mice, green bars). Data are presented as the mean ± SEM. ns, not significant (Tukey's multiple comparisons test). **(B)** Tail-flick latencies in a tail-flick test (beam intensity generating heat temperature at 80°C). Data are obtained from the same population in **(A)**. Data are presented as the mean ± SEM. ns, not significant (Tukey's multiple comparisons test). **(C)** Identification of laminar I/II in the lumber cord using calbindin-D28K immunoreactivity (see Figure 3C). Note that formalin-induced c-Fos-positive cells intensely localized in laminar I/II.

Supplementary Figure 3 PAG and BNST subdivisions without a significant increase in c-Fos expression after formalin injection. **(A)** Time schedule for the experiment. Female pups were subcutaneously injected with testosterone (Female + T) or sesame oil vehicle (Female [ctrl]) on the day of birth. In adulthood, mice were sampled 90 min after saline (S) or formalin (F) injection. **(B)** Schematic representation of the analyzed PAG area divided into the dorsomedial (DM-PAG), dorsolateral (DL-PAG), lateral (L-PAG), and ventrolateral (VL-PAG) divisions. DR, dorsal raphe. **(C)** The density of c-Fos positive cells in the DM-PAG and DL-PAG following saline or formalin injection into the let hindpaw. Data are obtained from males (5 mice for S and F), control females (8 mice for S and F), and females with neonatal testosterone administration (5 mice for S and F). ns, not significant by unpaired *t*-test. **(D)** Schematic representation of the analyzed anterior BNST area divided into the anteromedial (AM-BNST), anterolateral (AL-BNST), and anteroventral (AV-BNST) divisions. AC, anterior commissure. **(E)** The density of c-Fos positive cells in the AM-BNST following saline or formalin injection into the left hindpaw. Data are obtained from males (5 mice for S and F), control females (8 mice for S and F), and females with neonatal testosterone injection (5 mice for S and F).

Supplementary Figure 4 Systemic microglial ablation sex-independently suppresses formalin-induced pain responses. **(A)** Comparisons of the duration of licking behavior during phase 2 between mice fed a normal and PLX3397 (PLX)-containing diet. Data are obtained from males (9 mice for normal and 6 mice for PLX), control females (5 for normal and 11 for PLX), and females with neonatal testosterone administration (10 for normal and 6 for PLX). Data are the same as in Figures 6C, E, G. ^*^*p* < 0.05; and ^**^*p* < 0.01 (unpaired *t*-test). **(B)** Comparisons of PLX-induced decrease in the phase 2 licking duration. The duration of licking behavior in individual PLX-treated mice was subtracted from the average licking duration in each mice under the normal condition. ns, not significant (Tukey's multiple comparisons test).
