# Supplementary figures and images for: Neonatal testosterone exposure alleviates female-specific severity of formalin-induced inflammatory pain in mice
Source: Front Neural Circuits. 2025 Jul 2;19:1593443. doi: 10.3389/fncir.2025.1593443 (PMC12263906; doi:10.3389/fncir.2025.1593443)

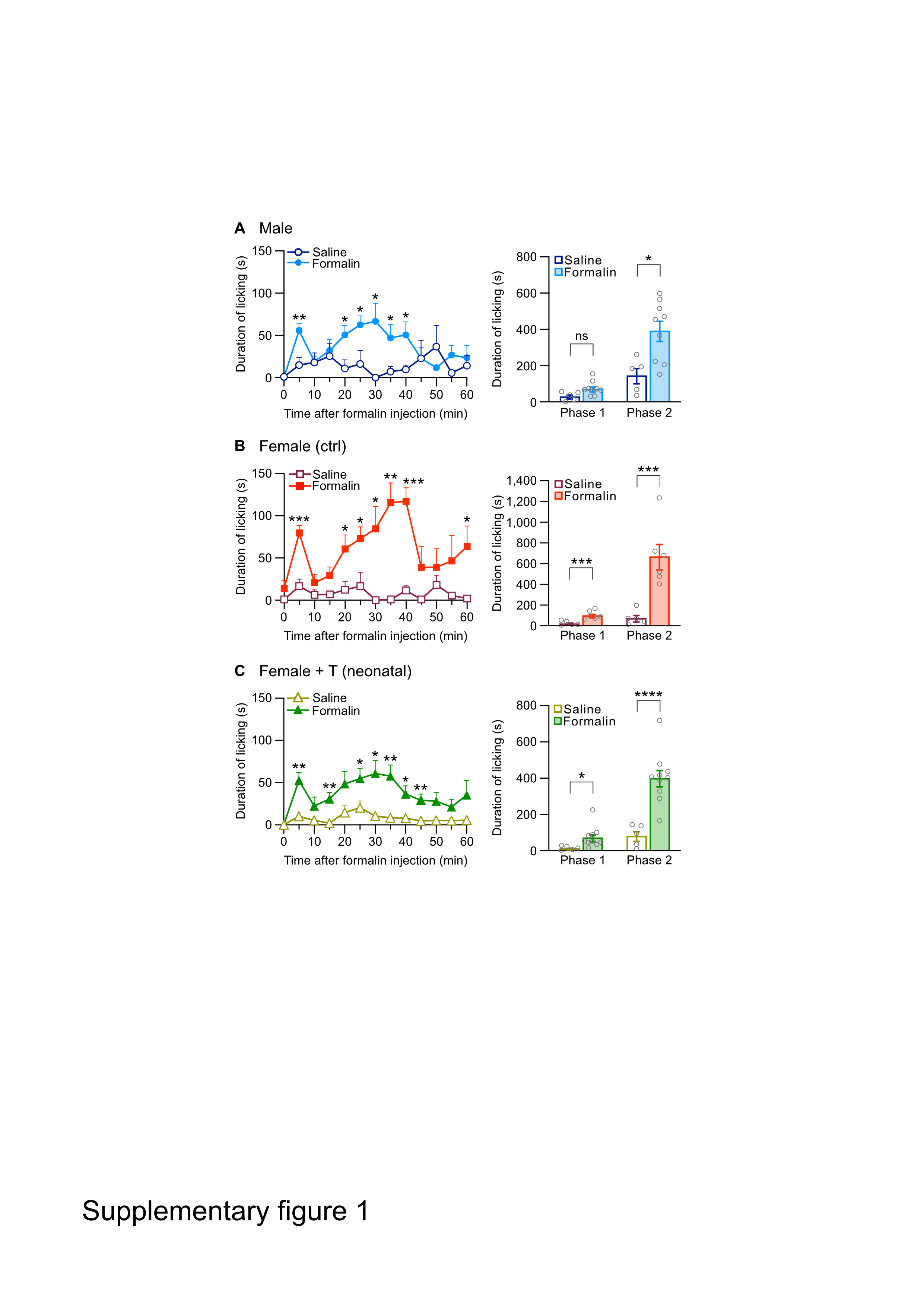

Supplement: Supplementary Figure 1 — Intraplantar formalin injection increases pain responses. (A) Male pain responses to intraplantar saline or formalin injection. Left graphs, the time course of the licking duration after intraplantar saline (5 mice) or formalin injection (9 mice). Right graphs, comparisons of the duration of licking behavior during phases 1 and 2. Data are presented as the mean ± SEM. Data for formalin-administered males are the same as in Figure 1B. *p < 0.05; and **p < 0.01 (left graphs, Tukey's multiple comparisons test; and right graphs, unpaired t-test). ns, not significant. (B) Female pain responses to intraplantar saline (5 mice) or formalin injection (5 mice). Data for formalin-treated females are the same as in Figure 1B. *p < 0.05; **p < 0.01; and ***p < 0.001. (C) Pain responses of females with neonatal testosterone administration to saline (5 mice) or formalin injection (10 mice). Data for formalin-treated mice are the same as in Figure 1B. *p < 0.05; **p < 0.01; and ****p < 0.0001. [file Image_1.tiff]

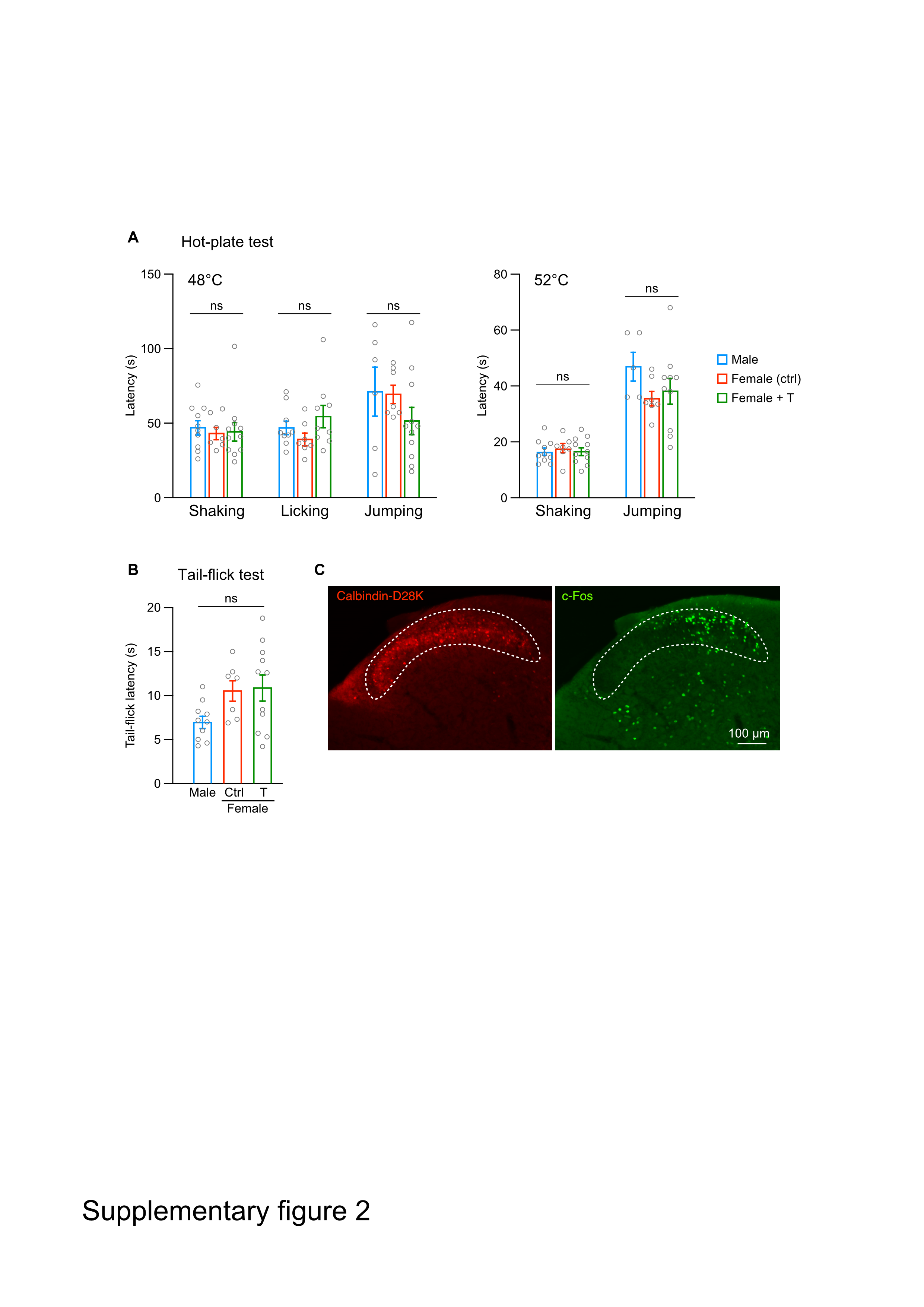

Supplement: Supplementary Figure 2 — Thermal pain responses are similar among males, control females, and females with neonatal testosterone administration. (A) Latencies to different pain responses at 48°C (left graphs) and 52°C (right graphs) in a hot-plate test. Data are obtained from males (10 mice, blue bars), control females with neonatal sesame oil administration (Female [ctrl], 7 mice, red bars), and females with neonatal testosterone administration (Female + T, 11 mice, green bars). Data are presented as the mean ± SEM. ns, not significant (Tukey's multiple comparisons test). (B) Tail-flick latencies in a tail-flick test (beam intensity generating heat temperature at 80°C). Data are obtained from the same population in (A). Data are presented as the mean ± SEM. ns, not significant (Tukey's multiple comparisons test). (C) Identification of laminar I/II in the lumber cord using calbindin-D28K immunoreactivity (see Figure 3C). Note that formalin-induced c-Fos-positive cells intensely localized in laminar I/II. [file Image_2.tiff]

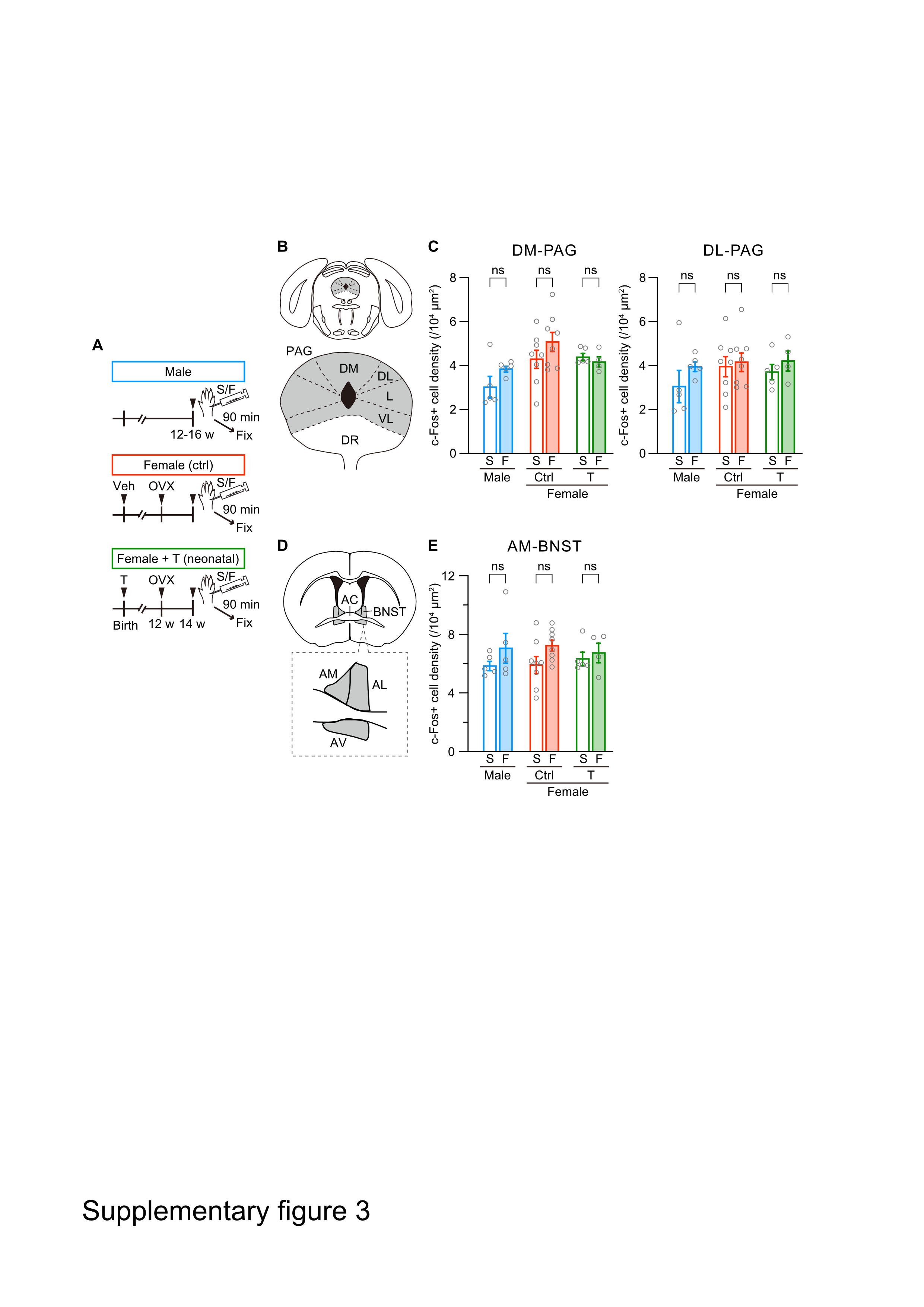

Supplement: Supplementary Figure 3 — PAG and BNST subdivisions without a significant increase in c-Fos expression after formalin injection. (A) Time schedule for the experiment. Female pups were subcutaneously injected with testosterone (Female + T) or sesame oil vehicle (Female [ctrl]) on the day of birth. In adulthood, mice were sampled 90 min after saline (S) or formalin (F) injection. (B) Schematic representation of the analyzed PAG area divided into the dorsomedial (DM-PAG), dorsolateral (DL-PAG), lateral (L-PAG), and ventrolateral (VL-PAG) divisions. DR, dorsal raphe. (C) The density of c-Fos positive cells in the DM-PAG and DL-PAG following saline or formalin injection into the let hindpaw. Data are obtained from males (5 mice for S and F), control females (8 mice for S and F), and females with neonatal testosterone administration (5 mice for S and F). ns, not significant by unpaired t-test. (D) Schematic representation of the analyzed anterior BNST area divided into the anteromedial (AM-BNST), anterolateral (AL-BNST), and anteroventral (AV-BNST) divisions. AC, anterior commissure. (E) The density of c-Fos positive cells in the AM-BNST following saline or formalin injection into the left hindpaw. Data are obtained from males (5 mice for S and F), control females (8 mice for S and F), and females with neonatal testosterone injection (5 mice for S and F). [file Image_3.tiff]

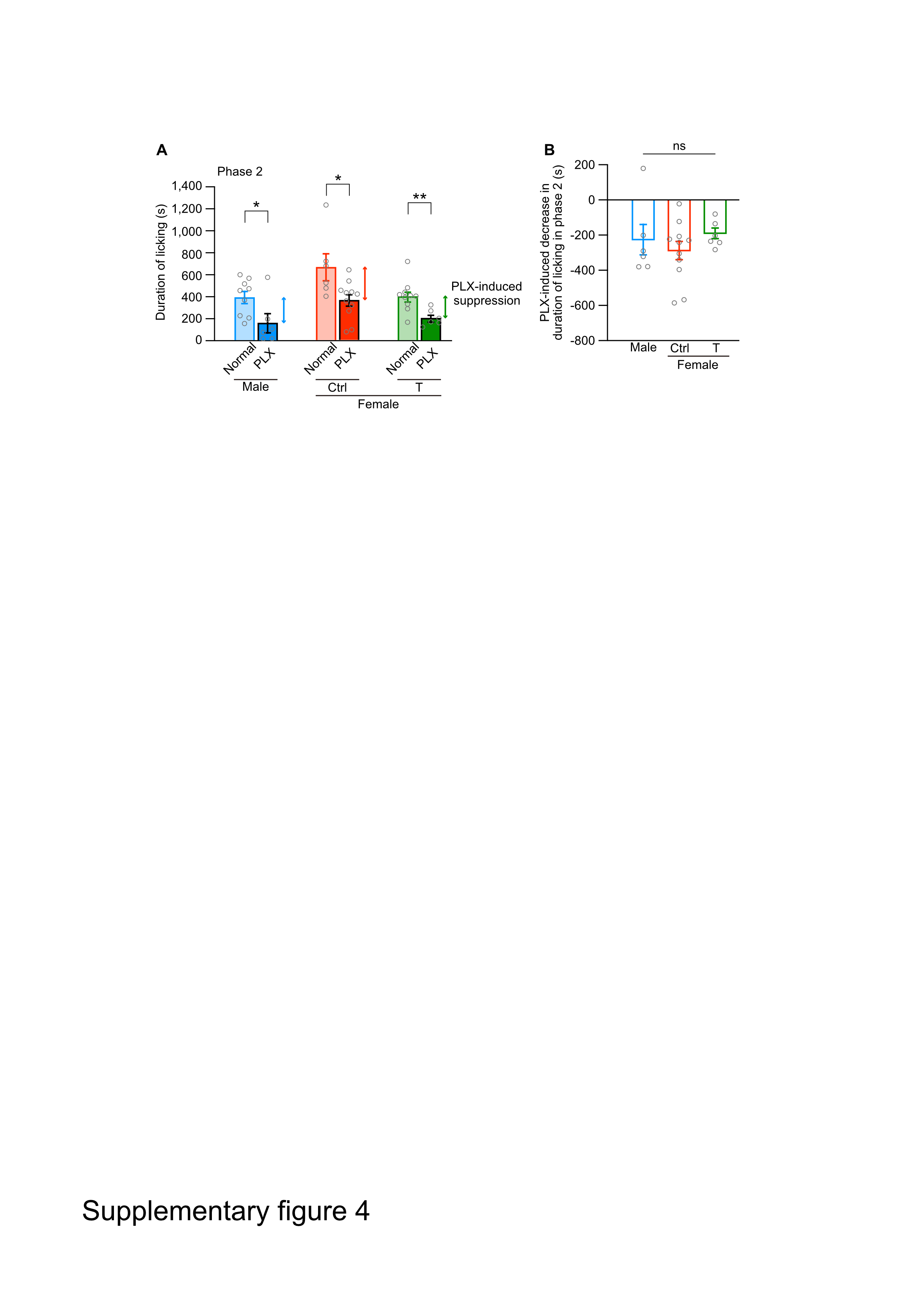

Supplement: Supplementary Figure 4 — Systemic microglial ablation sex-independently suppresses formalin-induced pain responses. (A) Comparisons of the duration of licking behavior during phase 2 between mice fed a normal and PLX3397 (PLX)-containing diet. Data are obtained from males (9 mice for normal and 6 mice for PLX), control females (5 for normal and 11 for PLX), and females with neonatal testosterone administration (10 for normal and 6 for PLX). Data are the same as in Figures 6C, E, G. *p < 0.05; and **p < 0.01 (unpaired t-test). (B) Comparisons of PLX-induced decrease in the phase 2 licking duration. The duration of licking behavior in individual PLX-treated mice was subtracted from the average licking duration in each mice under the normal condition. ns, not significant (Tukey's multiple comparisons test). [file Image_4.tiff]
